# Neuroprotective Effects of Ischemic Preconditioning on Global Brain Ischemia through Up-Regulation of Acid-Sensing Ion Channel 2a

**DOI:** 10.3390/ijms11010140

**Published:** 2010-01-12

**Authors:** Yifeng Miao, Weiqiao Zhang, Yuchang Lin, Xiaojie Lu, Yongming Qiu

**Affiliations:** 1 Department of Neurosurgery, Ren Ji Hospital, Shanghai Jiao Tong University School of Medicine, Shanghai 200127, China; E-Mail: yifengmwx@hotmail.com (M.Y.); 2 Nanjing Medical University, Affiliated Wuxi No. 2 Hospital, Neuroscience Center, Wuxi, China; E-Mails: zhang951154@sina.com (Z.W.); linyuchangwx@hotmail.com (L.Y.)

**Keywords:** acid-sensing ion channel 2a, ischemic preconditioning, global brain ischemia, neuroprotection

## Abstract

Transient forebrain or global ischemia induces cell death in vulnerable CA1 pyramidal neurons. A brief period of ischemia, *i.e.*, ischemic preconditioning, affords CA1 neurons robust protection against a subsequent, more prolonged ischemic challenge. Using the four-vessel occlusion model, we established an ischemic preconditioning model in which rodents were subjected to 3 min of sublethal ischemia 48 h before a 15 min lethal ischemia. We showed that preconditioning attenuated the ischemia-induced neural cell death and DNA fragmentation in the hippocampal CA1 region. RT-PCR and western blot analysis showed that preconditioning prior to an ischemic insult significantly increased ASIC 2a mRNA and protein expression in comparison to the ischemic insult alone (p < 0.01). These findings implicate a new role of ASIC 2a on endogenous neuroprotection from ischemic insult.

## 1. Introduction

Ischemic stroke, which results from cardiac arrest, cerebral arterial occlusion, or severe vasospasm after subarachnoid ischemia, causes devastating damage to the brain and represents a serious global health problem [1–4]. During ischemia, oxygen depletion forces the brain to switch to anaerobic glycolysis. The accumulation of lactic acid as a byproduct of glycolysis together with the production of protons by ATP hydrolysis cause a drop in the pH of the ischemic brain. Consequently, tissue pH typically falls to 6.5–6.0 during ischemia under normoglycemic conditions, and it can fall below 6.0 during severe ischemia or under hyperglycemic conditions [5–7]. Nearly all *in vivo* studies indicate that acidosis aggravates ischemic brain injury [8,9]. On the other hand, a short period of sublethal ischemia can induce tolerance in neurons to subsequent, more prolonged ischemia; a phenomenon known as ischemia tolerance [10–12]. Currently, the molecular mechanisms underlying both ischemic cell death and ischemia tolerance are largely unknown. Understanding the molecular basis of these phenomena is likely to provide new therapeutic strategies for this devastating neurological problem.

Acid-sensing ion channels (ASICs) are members of the Deg/ENaC superfamily of amiloride-sensitive ion channels [13–16]. To date, six ASIC subunits have been identified in mammalian systems. Four of these, ASIC 1a, ASIC 1b, ASIC 2a, and ASIC 3, form functional channels with distinct kinetics, conductance properties, pH sensitivities, and expression patterns [17–19]. A modulatory subunit, ASIC 2b, does not form a functional channel, but instead alters the properties of the other subunits [20]. The most recently cloned subunit, ASIC 4, is not activated by any known ligand and may thus also play a modulatory role [21]. When co-expressed heterologously, several subunits have been shown to associate into heteromultimeric channel complexes with properties distinct from those of homomultimeric channel complexes [22].

ASICs are expressed throughout the mammalian nervous system. Those in sensory neurons in the periphery have been implicated in the perception of pain during tissue acidosis [23]. The presence of ASICs in the brain suggests that these channels may have functions beyond nociception [8,24,25]. In particular, transcripts for ASIC 2a have been detected predominantly in the brain [26,27]. When expressed in heterologous systems, this homomultimeric channel is activated half-maximally at pH_0.5_ = 4.4 and conducts a transient, sodium-selective current. Mutation of residue Gly 430 to a bulky amino acid increases pH_0.5_ to 6.7, abolishes inactivation, and causes cell death. Mutation of the same residue in C. elegans degenerins causes neurodegeneration.

Because tissue acidosis accompanies ischemia, this study hypothesized that ASICs might play a role in mediating ischemic tolerance and the cellular responses to an ischemic insult. To test this hypothesis, western blot and RT-PCR were used to assess the expression of ASIC 2a after global ischemia and ischemic preconditioning. Our results show that ASIC 2a expression increases in the hippocampus after global ischemia, and that ischemic preconditioning can further increase ASIC 2a expression.

## 2. Results and Discussion

### 2.1. Physiological variables

We found that during brain ischemia there was a slight decrease in the pH in the Isch group, but this was not significantly different from the other experimental groups. In addition, the experimental groups did not differ with respect to the pre-, intra-, or post-ischemia blood pressure, hemoglobin, hematocrit, or serum glucose level (Table 1).

### 2.2. Cresyl violet stain

**Figure 1.**
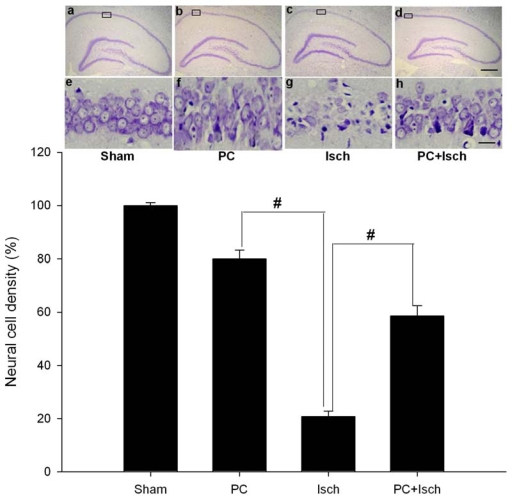
Effect of ischemic preconditioning on ischemia-induced neuronal cell loss in hippocampal CA1 regions. Animals were subjected to preconditioning (PC), global ischemia (Isch), or preconditioning followed by ischemia (PC + Isch), by the four-vessel occlusion paradigm (PC, 3 min, 48 h before Isch, 15 min), followed by reperfusion. Five days later, brains were fixed with paraformaldehyde followed by preparation of coronal sections from paraffin-embedded brains and subsequent staining with cresyl violet to determine cell survival in neuronal layeres of the hippocampi (n = 5). The boxed areas of CA1 subfield are shown at higher magnification, and the number of viable neurons in these areas was counted. Images of hippocampi at lower magnification (× 40) are a – d, and images at higher (× 400) are e – h. Error bar, 30 um. Data are mean ± S.D. # p < 0.01 by nonparametric ANOVA followed by Dunn’s analysis comparing with ischemic rats.

We examined whether preconditioning was associated with an increase in neuronal cell survival in the hippocampal CA1 region after ischemia. CA1 pyramidal cells in sham animals showed round and pale stained nuclei under cresyl violet staining (Figure 1a, 1e). In contrast, five days after lethal ischemia, most CA1 pyramidal cells were shrunken with pyknotic nuclei (Figure 1c, 1g). Sublethal ischemic insult alone also induced neural cell death, albeit less (Figure 1b, 1f). Interestingly, in the case of an ischemic insult, neuronal density was significantly increased by preconditioning ischemia compared to the ischemic insult alone (p < 0.01, Figure 1d, 1h). The number of surviving pyramidal cells in the CA1 region after a single ischemic insult and after preconditioning followed by an ischemic insult were 20.7 ± 2.1 and 58.6 ± 3.8% of those in the sham operation, respectively (Figure 1, a–h).

### 2.3. Tunel stain

**Figure 2.**
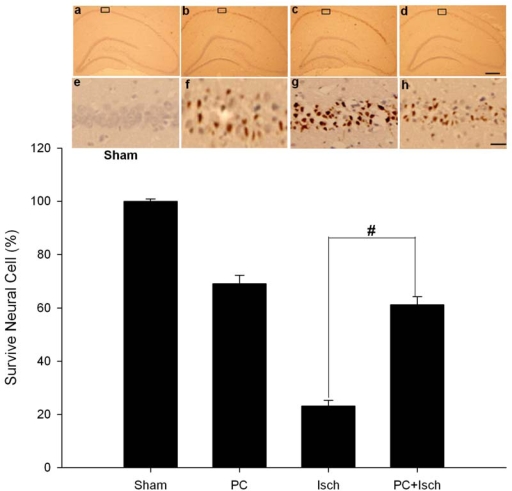
Effect of ischemic preconditioning on ischemia-induced neuronal apoptosis in hippocampal CA1 regions. Animals were subjected to preconditioning (PC), global ischemia (Isch), or preconditioning followed by ischemia (PC + Isch), by the four-vessel occlusion paradigm (PC, 3 min, 48 h before Isch, 15 min), followed by reperfusion. Seven days later, animals were killed and coronal sections (18 mm) of fresh-frozen rat brain were cut by cryotome. Sections were fixed in 4% paraformaldehyde for 60 min at room temperature and processed for TUNEL nuclear staining (n = 5). The boxed areas of CA1 subfield are shown at higher magnification, and the number of viable neurons in these areas was counted. Images of hippocampi at lower magnification (×40) are a–d, and images at higher (×400) are e–h. Error bar, 30 um. Data are mean ± S.D. # p < 0.01 by nonparametric ANOVA followed by Dunn’s analysis comparing with ischemic rats.

To examine DNA fragmentation in neurons undergoing apoptosis, we used TUNEL staining to label brain sections from sham and experimental rats seven days after ischemia. In sections from sham brains, TUNEL labeling was undetectable in the CA1 region (Figures 2a, 2e). Global ischemia induced a marked increase in the incidence of TUNEL—positive CA1 neurons (Figures 2c, 2g). Preconditioning significantly blocked ischemia-induced DNA fragmentation indicated by TUNEL (p < 0.01, Figures 2d, 2h).

### 2.4. RT-PCR

**Figure 3.**
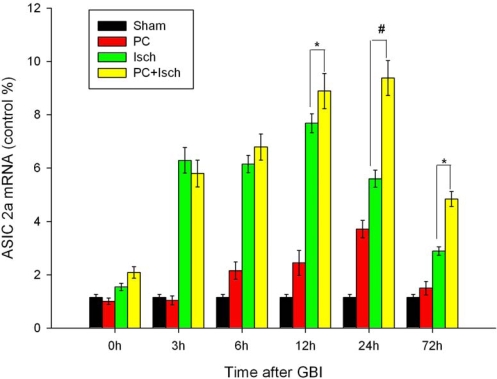
RT-PCR analysis of ASIC 2a mRNA expressions in hippocampal CA1 regions. Animals were subjected to preconditioning (PC), global ischemia (Isch), or preconditioning followed by ischemia (PC + Isch), by the four-vessel occlusion paradigm (PC, 3 min, 48 h before Isch, 15 min), followed by reperfusion. Rats were decapitated at 0 h, 3 h, 6 h, 12 h, 24 h or 72 h after reperfusion. Extracts from the hippocampi of the rats and sham controls were subjected to qualitative RT-PCR analysis. Data are mean ± S.D. (n = 5). * p < 0.05, # p < 0.01 by nonparametric ANOVA followed by Dunn’s analysis comparing with ischemic rats.

The time-course of ASIC 2a mRNA expression (as measured by quantitative RT-PCR) in the hippocampal CA1 region for all experimental groups is shown in Figure 3. We found that global ischemia induced a marked increase in ASIC 2a mRNA within three hours of ischemia. Expression was maximal at 12 h and diminished within 72 h. Preconditioning prior to the ischemic insult significantly up-regulated ASIC 2a mRNA compared to the ischemic insult alone (p < 0.01 at 24 h and p < 0.05 at 12 or 72 h, Figure 3). The increase in ASIC 2a mRNA expression in the hippocampal CA1 region in the preconditioned group (PC + Isch) was maximal at 24 h and persisted even at 72 h.

### 2.5. Western blot

**Figure 4.**
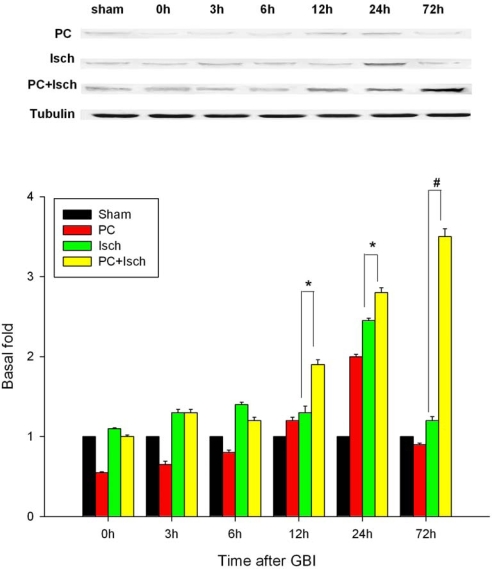
Western blotting analysis of ASIC 2a activations and expressions in hippocampal CA1 regions. Animals were subjected to preconditioning (PC), global ischemia (Isch), or preconditioning followed by ischemia (PC + Isch), by the four-vessel occlusion paradigm (PC, 3 min, 48 h before Isch, 15 min), followed by reperfusion. Rats were decapitated at 0 h, 3 h, 6 h, 12 h, 24 h or 72 h after reperfusion. Extracts from the hippocampi of the rats and sham controls were subjected to Western blotting with anti-ASIC 2a protein. Data are mean ± S.D. (n = 5). * p < 0.05, # p < 0.01 by nonparametric ANOVA followed by Dunn’s analysis comparing with ischemic rats.

Western blot was used to assess the effect of global ischemia and ischemic preconditioning on ASIC 2a protein expression (Figure 4). Lysates prepared from the hippocampi of sham, ischemic and preconditioned rats (both PC and PC + Isch) were probed with an antibody to ASIC 2a. Little protein was detected in the sham or PC group tissue. ASIC 2a protein was up-regulated after ischemia with expression being maximal at 24 h and then decreasing gradually. Preconditioning before the ischemic event significantly increased expression of the ASIC 2a protein compared to ischemia alone (p < 0.01 at 72 h, p < 0.05 at 12 h and 24 h, Figure 4). The expression of ASIC 2a protein in PC + Isch group increased over time and was maximal at 72 h.

### 2.6. Discussion

In the present studies, we showed that lethal ischemic insult for 15 min caused abundant neuronal cell death and apoptosis in the hippocampal CA1 region. However, when the animals were pretreated with preconditioning for 3 min two days before the lethal ischemic insult, the above neuronal injury was significantly attenuated. Further studies showed that global ischemia up-regulated the expression of ASIC 2a, a finding that is in accordance with a previous study [27]. We also found that preconditioning increased the ASIC 2a mRNA and protein to a level higher than that induced by a single ischemic insult. These findings might provide some clues towards the mechanism underlying ischemia tolerance and aid in the search of clinical therapies for stroke using endogenous neuroprotective methods.

It is well known that acidosis is a prominent event in ischemia [9,30]. Therefore, the numerous effects of acidosis in the ischemic brain have received extensive study. Recently, a new class of ion channels, the acid-sensing ion channels (ASICs), have been found to be present throughout the brain tissue [8,25]. In addition, previous studies suggested that the brain’s response to ischemia may be mediated by a combination of several mechanisms, many of which remain to be elucidated [31]. Therefore, our study explored the possible involvement of ASICs in the ischemic response. To this end, that ASIC2a can play the role of neuroprotectionwe used western blot and RT-PCR to assess the expression of the ASIC 2a mRNA and protein in normal and ischemic rat brains. Our study showed that global ischemia up-regulated the expression of ASIC 2a in the hippocampal CA1 region. These results are consistent a previous study that found that the expression pattern of ASIC 2a was similar to that of antiapoptotic proteins Bcl-2 and Bcl-w, results that suggested a protective role for this channel [27].

Immediately after global ischemia, the extracellular pH of the brain decreases from 7.2 to 6.5, a level sufficiently low to activate ASIC 1a and ASIC1a/ASIC 2a comprising channels [7]. The up-regulation of ASIC 2a in the ischemic brain in response to prolonged acidosis may alter the composition of existing channels. Studies performed in heterologous systems suggest that the effect of such a change might be to make those cells expressing ASIC 2a less responsive to acidosis. The pH of maximal activation (pH0.5) of the ASIC 1a channels is 6.2, whereas ASIC1a/ASIC 2a and ASIC 2a channels show a pH0.5 of 4.8 and 4.35, respectively. Association with ASIC 2a also slows the inactivation kinetics of ASIC 1a and increases its selectivity for Na^+^ over Ca^2+^ [32–34]. This might be significant in the context of ischemia, in which intracellular Ca^2+^ overload has been implicated as a primary mechanism of neuronal injury [35–37].

Ischemic preconditioning affords robust protection of CA1 neurons against a subsequent severe ischemic challenge [38]. However, the molecular mechanisms underlying ischemic tolerance are only partially understood. Because the ASIC 2a channels seem to have a neuroprotective ability, we hypothesized that preconditioning, an experimental protocol known to induce endogenous neuroprotection, might up-regulate the ASIC 2a protein. Using the four-vessel occlusion model, we found that preconditioning attenuated neural cell death and apoptosis (Figures 1, 2). In addition, RT-PCR and western blot analysis showed the preconditioning followed by an ischemic event significantly increased the ASIC 2a mRNA and protein expression when compared to a single ischemic insult (p < 0.01, Figures 3, 4).

A decrease in pH is a central metabolic consequence of ischemia, but one whose impact on cell survival is complicated and controversial. Some studies have found that severe acidosis (pH 5.5 to 6.0) after prolonged ischemia causes pan necrosis. In contrast, mild acidosis after transient ischemia does not appear to contribute to selective neuronal necrosis but does have a neuroprotective effect instead. This might be due to the fact that extracellular pHs of 6.5 to 7.0 suppress NMDA channel currents, thereby attenuating the injurious effects of glutamate [39]. Therefore, the acid-sensing ion channels might provide another possible direction from which to explore the impact of acidosis on the outcome of ischemia and how to protect the brain from ischemic injury.

## 3. Experimental Section

### 3.1. Animals

Age-matched adult male Sprague-Dawley rats weighing 200–220 g (Shanghai JiaoTong University Animal Laboratories) were maintained in a temperature- and light-controlled environment with a 14/10-h light/dark cycle. All subjects were treated in accordance with the principles and procedures of the Animal Care and Experimental Committee of the School of Medicine of Shanghai JiaoTong University.

### 3.2. Ischemic preconditioning and global ischemia

Animals were subjected to preconditioning (PC), global ischemia (Isch), or preconditioning followed by ischemia (PC + Isch) by the four-vessel occlusion paradigm followed by reperfusion, as described previously [28,29]. Preconditioning consisted of a three-minute occlusion that took place 48 h prior to the global ischemic event, which consisted of a 15-minute occlusion. One day before clamping the carotids for PC or Isch, the vertebral arteries were cauterized and the carotids were exposed. Sham surgeries consisted of subjecting animals to the same anesthesia and surgical procedures, except that the carotid arteries were not occluded. Sham, PC, and PC + Isch animals were sacrificed at times corresponding to those of the Isch animals. During surgery, body temperature was monitored and maintained at 37.5 ± 0.5 °C with a rectal thermistor and a heat lamp. Monitoring continued until the animals had fully recovered from anesthesia. Animals that failed to show complete loss of the righting reflex 2 min after occlusion were excluded from the study. The mortality after ischemia was ~10%.

### 3.3. Histological analysis

Neuronal cell loss was assessed by histological examination of the dorsal hippocampus (CA1 region) of brain sections stained with cresyl violet as previously described [29]. Animals were sacrificed five days after the sham, Isch, PC, or PC + Isch surgeries. To this end, animals were deeply anesthetized with halothane and fixed by transcardiac perfusion with ice-cold 4% paraformaldehyde in PBS (0.1 M, pH 7.4). Brains were removed and immersed in fixative. Coronal sections (15 um) were cut at the level of the dorsal hippocampus with a cryotome and stained with cresyl violet. The number of non-apoptotic pyramidal neurons per 250 um length of the medial CA1 region was counted under a light microscope at ×40 magnification. Four sections were counted from each rat, with each experimental group consisting of five rats.

### 3.4. In Situ labeling of DNA fragmentation by TUNEL

To detect DNA fragmentation in degenerating neurons, animals were sacrificed seven days after reperfusion, and coronal sections (18 μm) of freshly-frozen rat brain were cut using a cryotome. Sections were fixed in 4% paraformaldehyde for 60 min at room temperature and processed for TUNEL nuclear staining using an *in situ* cell death detection kit (Roche Molecular Biochemicals and Molecular Probes; manufacturer’s instructions were followed). Images were viewed under a Nikon ECLIPSE TE300 fluorescence microscope and acquired with a SPOT RT CCD-cooled camera equipped with diagnostic software version 3.0. Imaging settings were kept constant across experimental and control groups. TUNEL-positive cells were identified directly by the fluorescence signal of incorporated fluorescein-dUTP. Cells in 32 fields sampled from the CA1 region of five animals were scored.

### 3.5. Reverse transcription—Polymerase chain reaction

Comparison of mRNA levels for the ASIC 2a subunit was carried out at 0 h, 3 h, 6 h, 12 h, 24 h and 72 h after ischemic insult. Quantitative real-time RT-PCR with the ABI prism 7500 PCR^®^ Systems (Applied Biosystems, Ltd., USA.) was used to measure mRNA levels for ASIC 2a in the hippocampus. Tissue from the hippocampus was homogenized in Trizol reagent (Invitrogen, Carlsbad, CA) for extraction of total RNA, following the manufacturer’s protocol. In a sterile, RNase-free microcentrifuge tube, 2 μg of total RNA and 1 μL of 20 uM oligo(dT)_15_ primer were combined to a total volume of 15 μL. The tube was then heated at 70°C for 5 min to melt the secondary structure within the template. Next, the tube was cooled immediately on ice to prevent the formation of additional secondary structures. The tube was then spun briefly to bring the solution to the bottom of the tube. The following components: 5 μL of 5 × M-MLV Reaction Buffer, 1.25 μL of 10 mM dNTPs, 25 units of RNasin Ribonuclease Inhibitor, and 200 units of M-MLV RT RNAse H- (Promega, Madison, WI), were added to yield a total reaction volume of 25 μL. All reactants were mixed gently by flicking the tube and then incubated at 42 °C for 60 min before terminating the reaction at −20 °C. Expression of the β-actin gene acted as the housekeeping gene control. The following primer pairs were used for the rat ASIC 2a transcript:

ASIC 2a _Forward:5′-ATGTTTAACTCAGGCGAGGATG-3′
ASIC 2a _Reverse:5′-CCACGAAGGTCTGGAACCC-3′

Separate PCR reactions (25 μL) were conducted for each transcript and they comprised cDNA (2.0 μL), 12.5 μL of 2 × SYBR *Premi E Taq* (TaKaRa, Ltd., Japan), and 0.5 μL of 10 μM gene-specific forward and of 10 μM reverse primers. PCR conditions were optimized to 95 °C (30 s), followed by 40 cycles (45 s each) at 95 °C, 60 °C (5 s), and 72°C (30 s), completing the reaction at 37 °C for 30 seconds. Five animals were used in each group.

### 3.6. Western blotting

Western blots were used to measure ASIC 2a protein levels at each time point. The brain tissue (hippocampus CA1) was homogenized for 30 min at 4 °C in 200 mL of RIPA lysis buffer containing: 50 mM Tris-HCl pH 7.4, 150 mM NaCl, 1 mM EDTA, 1% Triton x-100, 1% sodium deoxycholate, 0.1% SDS, add 1 mM PMSF and protease inhibitor cocktail. The insoluble material was removed by centrifugation at 16,000 rpm at 4 °C for 30 min. Protein concentration in the supernatant was measured using BioRad Dc Protein Assay kits (cat# 500-0116, BioRad). Equal amounts of protein (60 μg) were loaded and separated by sodium dodecyl sulfate-polyacrylamide gel (SDS-PAGE) electrophoresis on a 10% polyacrylamide gel. After electrophoretic transfer of the separated polypeptides to an Immobilon-PVDF membrane (Millipore) at 300 mA for one hour on ice, the membranes were blocked with 5% nonfat milk in PBS. The membranes were then washed and incubated overnight with the primary antibodies with 5% nonfat milk in PBS at 4°C. The following primary antibody was used: rabbit polyclonal anti-ASIC 2a (cat# 77384-100 dilution 1:1,000, Abcam, Ltd., USA). After incubation with the primary antibody, the PVDF membranes were washed with a PBST dilution and incubated with the appropriate IRDye CW700-labeled goat anti rabbit IgG secondary antibody (dilution 1:5,000, Li-cor, USA) using 5% nonfat milk in PBS for one hour at room temperature. After two rinses and four washes with PBST, the membranes were scanned using the Odyssey infrared Imaging System (Li-cor, USA) for visualization of the bands. Membranes were stripped in strip buffer (6.35 mL of 1M Tris-HCl pH 6.8; 5 mL 20% SDS; 350 μL of 2-mercaptoethanol; 38.3 mL of distilled water). Tubulin (dilution 1:1,000, Sigma, USA) was used as the internal control. Five animals were used in each group.

### 3.7. Statistical analysis

The statistical significance was examined by ANOVA, followed by Dunn’s test. P < 0.05 was considered statistically significant.

## 4. Conclusions

Our studies showed that lethal ischemic insult for 15 min caused abundant neuronal cell death and apoptosis in the hippocampal CA1 region. However, when the animals were pretreated with preconditioning for 3 min two days before the lethal ischemic insult, the above neuronal injury was significantly attenuated. Further studies showed that global ischemia up-regulated the expression of ASIC 2a. We also found that preconditioning increased the ASIC 2a mRNA and protein to a level higher than that induced by a single ischemic insult. These findings might provide some clues towards the mechanism underlying ischemia tolerance and aid in the search of clinical therapies for stroke using endogenous neuroprotective methods.

## Figures and Tables

**Table 1.** Physiological variables did not significantly differ between experimental groups.

	**Sham**	**Isch**	**PC**	**PC + Isch**
Before Ischemia (n = 5)				
pH	7.44 ± 0.04	7.45 ± 0.07	7.43 ± 0.03	7.44 ± 0.05
pCO2 (mmHg)	35.6 ± 1.6	36.2 ± 4.2	38.2 ± 5.2	37.6 ± 4.9
pO2 (mmHg)	106.2 ± 10.9	110.2 ± 10.2	108.4 ± 8.3	105.4 ± 6.7
Glucose (mg/dL)	118.9 ± 7.4	121.1 ± 12.4	119.4 ± 7.0	129.9 ± 8.4
Hemoglobin (g/dL)	16.5 ± 1.2	15.6 ± 0.9	16.7 ± 0.5	16.2 ± 0.6
MABP (mmHg)	86.2 ± 7.1	85.3 ± 11.1	79.6 ± 12.3	84.2 ± 11.4
Temp rect (°C)	36.6 ± 0.3	36.7 ± 0.16	36.6 ± 0.21	36.6 ± 0.25

During Ischemia (n = 5)				
pH	7.41 ± 0.05	7.38 ± 0.12	7.39 ± 0.11	7.40 ± 0.08
pCO2 (mmHg)	41.2 ± 6.1	40.1 ± 3.5	41.8 ± 3.9	41.1 ± 5.1
pO2 (mmHg)	93.1 ± 5.6	98.1 ± 7.2	96.1 ± 5.4	99.2 ± 10.2
Glucose (mg/dL)	109.1 ± 5.6	104.7 ± 7.7	98.4 ± 10.4	116.9 ± 11.2
Hemoglobin (g/dL)	18.4 ± 2.1	19.1 ± 1.2	17.2 ± 5.1	19.2 ± 3.6
MABP (mmHg)	71.8 ± 8.6	75.1 ± 10.5	74.7 ± 8.7	79.2 ± 7.2
Temp rect (°C)	36.7 ± 0.12	36.9 ± 0.05	36.8 ± 0.03	36.8 ± 0.08

After Ischemia (n = 5)				
pH	7.41 ± 0.05	7.40 ± 0.03	7.39 ± 0.06	7.41 ± 0.03
pCO2 (mmHg)	38.0 ± 3.8	35.9 ± 4.2	40.1 ± 3.5	36.1 ± 1.8
pO2 (mmHg)	106.7 ± 8.4	115.8 ± 5.9	109.4 ± 9.2	116.4 ± 10.3
Glucose (mg/dL)	110.6 ± 7.9	109.2 ± 10.3	115.4 ± 13.2	117.9 ± 12.9
Hemoglobin (g/dL)	15.3 ± 1.1	16.4 ± 1.6	16.1 ± 1.7	15.7 ± 0.9
MABP (mmHg)	73.1 ± 12.3	77.3 ± 11.3	72.9 ± 4.9	73.1 ± 11.2
Temp rect (°C)	36.5 ± 0.11	36.9 ± 0.12	36.7 ± 0.05	36.9 ± 0.03

MABP: mean arterial blood pressure; Temp rect: rectal temperature. Values are means ± S.D., n = 5 in each group. All animals remained normothermic before, during and after ischemic injury. No significant differences across groups were observed with respect to arterial blood pressure and arterial blood gases.
